# A Family-Centered Intervention to Monitor Children's Development in a Pediatric Outpatient Setting: Design and Feasibility Testing

**DOI:** 10.3389/frhs.2021.739655

**Published:** 2021-11-16

**Authors:** Muneera A. Rasheed, Waliyah Mughis, Kinza Naseem Elahi, Babar S. Hasan

**Affiliations:** ^1^Centre for International Health, Department of Global Public Health and Primary Care, University of Bergen, Bergen, Norway; ^2^Department of Paediatrics & Child Health, Aga Khan University, Karachi, Pakistan; ^3^Institute of Professional Psychology, Bahria University, Karachi, Pakistan

**Keywords:** Care for Child Development, developmental monitoring, family-centered support, nurturing care, Survey of Well-Being of Young Children

## Abstract

The patient experience team at a private tertiary care hospital used the Theory of Change to design a family-centered developmental monitoring intervention, building on an ongoing initiative. The design entailed (i) a monitoring form: Survey of Well-Being of Young Children (SWYC) being an easy parent-report measure; (ii) family support intervention: the Care for Child Development module to enhance parent-child interactions; (iii) timing: utilizing wait time to also enhance families' experience; (iv) the service providers: psychology trainees as volunteers; and (v) reinforcement: by the pediatrician in the regular consultation health visit capitalizing on the established rapport with families. All families with children under 5 years 5 months 31 days of age in selected acute, complex, and developmental care clinics were eligible. Feedback from stakeholders indicated that the monitoring process was useful and imparted important information for parents and pediatricians, while the trainees felt the experience to be significant for their own learning. The authors conclude that the designed intervention model for a family-centric approach was acceptable and feasible. Key recommendations have been presented for further scale-up.

Early identification of children at risk of sub-optimal development or delay and subsequent interventions can lead to improved developmental outcomes (1). Hence, developmental monitoring at different times in a child's life (to be completed at least at 9, 18, and 30 months of age during a child's critical developmental period) for adequate referral is considered an integral practice in high-income settings, such as the USA (2). Despite a high burden in low- and middle-income countries (LMICs) with more than 250 million children not meeting their potential (3) and 1 in 6 of these children experiencing developmental difficulty (4), integrated developmental monitoring with appropriate referral is not a priority of the healthcare system as it requires continued efforts and engagement on the part of both the health care provider and the families (5–7). When available, screening is seldom followed by accessible evidence-based interventions. This was identified in a technical meeting by the World Health Organization (WHO) where the experts recommended broadening the scope of developmental monitoring to also include family-centered participatory support interventions ensuring nurturing care for all (4). Examples of family-centered interventions include the nurturing care interventions which aim to enable families to create an environment that meets the needs of young children i.e., nutrition and health, freedom from threats, and opportunities for early learning, through emotionally warm interactions (8). The experts have argued that these guidelines will support promotion of early development, acting as a preventive strategy for future developmental difficulties which is critical given the scale of the problem. The guidelines place the primary care providers in a unique position to promote children's development given their relationship with the family and being aware of the specific strengths built on regular contacts which can support children's development (9).

Sustainable integration of nurturing care into primary healthcare at scale requires partnerships not just between family and providers or between different providers but also broadly within and across sectors like public, private, and civil society (10, 11). Private healthcare providers are an important partner to tap into, especially in contexts where they are the major contributors. In Pakistan, the private sector covers up to 75% of the population's healthcare (12, 13) and only 2.8% of the country's budget is annually invested in healthcare, which is globally one of the lowest (14).

Pakistan has a substantial burden of children at risk for not attaining their developmental potential with high rates of maternal mortality (140/100,000), under-five mortality (69/1,000) and stunting (38%) (15). Moreover, no national data on provision of nurturing care practices at home for children under 3 years of age are available. Evidence suggests a loss of 20% in adult productivity if the risk is not mitigated through timely interventions (16). Similarly, epidemiological data related to childhood disability from Pakistan are limited but a few studies suggest physical disability to be its leading cause (17). Barriers such as social stigma and cultural norms, inadequate health infrastructure, and shortage of qualified professionals in early child development (ECD) prevent parents from seeking appropriate timely support for their differently abled child (17). There were reportedly only 54 qualified rehabilitation professionals in the country in 2016 (18). Children with disabilities face additional challenges and are denied admission to schools and parents may be advised to take their children to special schools (19).

The majority of the ECD and disability research and programmatic work in Pakistan is community-based in the public sector with scarce evidence of collaboration from the private health sector. A study with private outpatient clinics with mothers of young children found that counseling focused on promoting development were more engaging and helpful than the usual/standard care provided by pediatric consultants (20). However, the program was funded through a research grant, and scale-up of the innovation will likely remain dependent on philanthropy for further implementation. Given the financial adversity currently in the country, it is not a sustainable option. Long-run integration of family support practices within pediatric care to transform the development of millions of children requires context-specific and cost-effective approaches similar to social innovations taking into account not just technical feasibility but also market sustainability and economic viability of the population and the healthcare providers to ensure uptake (21). The nurturing care operational framework also recommends partnership with the private sector as one of the strategies to innovate and scale-up (22).

Examples of social innovations from similar contexts like India operate on these principles for a successful scale-up: designing interventions emphasizing a value for more rather than a perfectly designed model serving a few, utilizing and strengthening existing systems for reduced costs, and ensuring respect and experience of the families served (23). Additionally, literature from implementation sciences strongly suggests the use of a robust framework like Theory of Change (ToC) to design complex behavior change interventions involving multiple touch points and actors (24). The ToC methodology outlines how the intervention will work in real settings, describing the processes through which the change will happen and the assumptions inherent but specific to the context (25). World Health Organization (4) recommendations for a family-centered approach to developmental monitoring will require further guidelines on operationalization in primary care. Use of ToC to implement these guidelines following the principles of social innovation has not been tested yet.

Implementation of family-centered interventions requires additional effort to create a culture of family-centeredness in healthcare settings for sustainable behavior change (26). An ongoing initiative in the pediatric services at a tertiary hospital aiming to improve child and family experience outcomes with a focus on inpatient care (27) provided an excellent opportunity to test a model of family-centered developmental monitoring. The objective of this study was to develop and test the feasibility of integrating a family-centered developmental monitoring intervention as part of a larger initiative in a private pediatric care setting in Pakistan.

## Methods

### Setting

The study was conducted at a tertiary care teaching hospital and Joint Commission International-accredited hospital (JCIA) in Pakistan. Annually about 75,000 patients visit the pediatric outpatients' clinics. Major child specialties include but are not limited to: cardiopulmonary, neurology and rehabilitation, gastroenterology, endocrinology, nephrology, genetics, fetal and neonatal problems, and infectious diseases. Well-baby clinics are conducted, but developmental monitoring is yet to be established in the system. Rehabilitation services are available for children with needs under the section of neurology. A physician was under training in Canada for developmental pediatrics (the first in the hospital) during the course of the study to join as faculty in the coming year. The fee structure is comparable to other private tertiary health centers within the city. Since it is a private elite setting, relatively affluent families seek consultation. Data from previous work indicate about half of the mothers' accessing pediatric services have completed 10 years of education or more (28). Patients are primarily from the city and surrounding urban and rural areas within the province of Sindh. The physicians mostly communicate in the clinic in Urdu (the national language of the country). However, a significant majority of the families can also converse in English given it is the main administration language in the country and hence also used for hospital documentation. The implementation of the World Health Organization Global Disability Action Plan in Pakistan (GDAP) requires engagement of healthcare professionals and public-private institutional partnerships in order to provide appropriate awareness and rehabilitative access to patients and parents with ECD needs (29). The study was approved as a quality improvement project by the institutional Ethics Review Committee.

### Workflow

In the outpatient clinics, patients book their appointments online or by calling the hospital helpline. Patients are expected to arrive and register and pay the consultation fee 20 min prior to the appointment time. Next, the child/parents are then called into the assessment room where a nurse records, in the patient file, the child's height, weight, temperature, blood pressure, risk of fall, known allergies, and any prescribed medications. The patient file is shifted to the file tray outside the physician's consulting room and parents/families are then requested to sit in the waiting area until they are called to meet with the consultant. Average time spent in the clinic from start to finish ranges from 75 to 120 min. Waiting time varies between physicians, ranging from 40 to 80 min which is an opportunity to engage patients in an educational activity. No toys, play equipment, or books are currently available in the waiting area for children and their parents to utilize during long waiting times. One physician on average may see up to 112 patients per month (ranging from 55 to 235 patients monthly per physician, depending on specialty).

### Research Design

This feasibility study (30) was conducted as a quality improvement project (31) in the pediatric service line at the hospital for improved patient and family experience of care. The primary considerations of feasibility were physical space/design, human and material resources, and physician follow-up with patients identified to be at developmental risk.

### Sample

The inclusion criteria comprised all the children (patients) who visited the 10 selected physicians in the outpatient department (OPD), with an age range of 1 month, 0 days to 65 months, 31 days. The physicians were selected to cover a broad range of disease: acute care (6 out of 18 general pediatricians), complex care (3 of 6 pediatric cardiologists), and developmental care (1 of 2 specialists). Prior permission from physicians was sought to complete developmental screening with their patients. Permission was obtained from the physicians and the psychologist to conduct research with their patients. The parents of the patients were briefed about the purpose of the survey and verbal consent was obtained before being interviewed.

### Intervention Design

**Figure 1:**
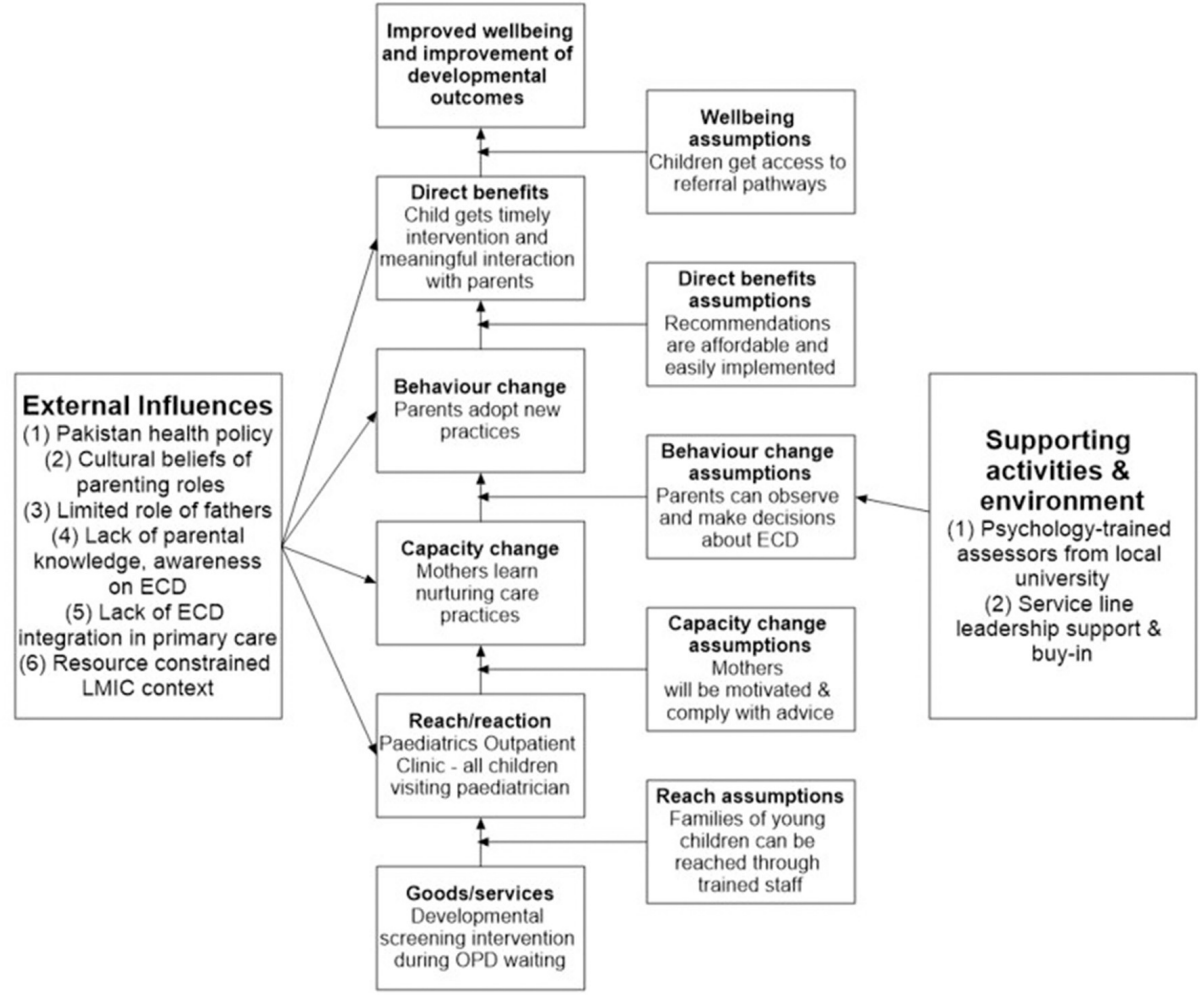
Theory of change model for integration of family-centered developmental monitoring. ECD, early child development; LMIC, low-and middle-income countries; OPD, outpatient department.

The implementation opportunity was identified by an ECD researcher, practicing as a developmental psychologist and also serving as the Director Patient Experience of Care in the service line (first author). The study was conducted between August 2019 and February 2020 as part of the larger initiative in place since October 2017 allowing the focus to shift to family and patient experience in the outpatient department. The intervention model was designed using the ToC guided by the following principles: ensure value addition for all stakeholders, leverage existing strengths, and keep it simple and cost-effective yet comfortable while being grounded in science at the same time. We used a backward mapping approach with a main focus on also intervening for the assumptions we were making as part of the strategy as recommended by Mayne (32), e.g., it required physician engagement which meant leadership buy-in and support. Hence, we started this service, once the larger initiative was fairly established. Moreover, we needed engaged delivery staff with minimal financial implications. Based on our previous experience, psychology trainees were selected, and we had a memorandum of understanding with the University to credit the trainees with internship hours. The ToC was developed after thoughtful considerations by the patient experience team about how the intervention would work in the context. It was realized that the intervention had to be framed to also benefit family experience to gain leadership buy-in as developmental disabilities may not be a priority in a system burdened with physical diseases. Hence, it was housed in the Office of Patient Experience. The context for assumptions around different intervention components was analyzed based on the observations and experience of the team members which included the Director Patient Experience of Care (first author) and the Service Line Chief (last author) (Figure 1).

#### Developmental Monitoring Tool

Given the intervention was meant to enhance the parental role in children's development, it was important to ensure any child who could potentially benefit from just a conversation with the family was not missed. A tool was needed that was feasible to be completed by parents and for subsequent use by primary care providers. The Survey of Well-being of Young Children (SWYC) is a freely available, first level developmental-behavioral screening tool developed by researchers at The Floating Hospital for children under 65 months, 31 days of age at Tufts Medical Center (33). The form is simple, provides a holistic screening across developmental domains, emotional & behavioral adjustment, and also environmental factors emphasizing the role of the context. The SWYC has age-specific forms with a total of 12 different forms for 12 age groups.

#### The Family Support Intervention

The psychosocial support intervention was based on principles of the *Care for Child Development* (CCD) module (34). The module includes messages to enhance nurturing parent-child interactions using developmentally appropriate play activities. The addition of this intervention was meant to shift the focus from a deficit model to nurturing the strengths of the families, enabling them to provide early learning and stimulation opportunities to their child. All families can positively contribute toward the development of their child and may just need minimum coaching. The first author is a certified trainer for implementing the CCD module and has successfully used it as an intervention for in-patient children and families in collaboration with psychology trainees as the delivery staff (28).

#### Timing for Administering the Developmental Monitoring Form

Pediatrician clinics are busy and adding on monitoring forms may be seen as a burden affecting engagement with the intervention. However, an opportunity of utilizing the long wait between clinical assessment and the appointment was identified—which was a major main pain point for families (35). When wait time cannot be reduced, some hospitals have utilized these times to encourage play (36) and reading (37) through volunteers for families of young children for a productive use of time. A study in Jamaica in the waiting area in primary care implemented a video-based ECD intervention led by community health workers. The results showed benefits for parental knowledge and child development when compared to controls (38). Moreover, utilizing the wait time, the family experience of an otherwise cumbersome wait, can improve significantly. Patient and family experience is increasingly being recognized specifically within the private sector as a key strategy for enhancing patient satisfaction with the services resulting in buy-in from hospital leadership in patient experience strategies (39).

#### Delivery Staff

The country has a handful of developmental pediatricians (40) with few training opportunities in residency programs (41) suggesting the urgency of initiating intervention models with opportunities to iteratively refine during implementation. The limited supply of professionals qualified in ECD is reflective of the low demand from parents of young children to monitor development. Such non-medical services are not usually valued enough for families to pay additionally for them. Moreover, such a service would be seen as burdensome by most families resulting in low uptake if initiated in silo (42). Developmental screening though important is not done routinely as not enough demand has been created. Hence, it is not seen as valuable by patients and physicians who do not see it generating revenue which is a crucial aspect for the survival of the services especially in a private center (43). Making this part of the regular clinic would mean identifying an additional cadre of care providers but without major financial investment. The cadre identified was psychology trainees from a local university who had a memorandum of understanding (MOU) with the service line. The trainee-led model was successfully implemented in the inpatient care facility whereby they received credit for internship hours spent at the hospital and were supervised which was an added incentive (28). A similar model would give the pediatrician additional, important information that adds value to that regular clinic visit for health issues at no cost to the practice. The developmental monitoring and support could be henceforth feasibly integrated by the pediatrician within their routine consultation services. The recommendations for follow-up for parents when reinforced by the pediatrician who are seen to be more trustworthy by families could enhance families' trust in the process (44). This would also allow pediatricians to assess the effect of child health issues on their functioning. Thus, creating value around child development may increase engagement and parental ownership.

#### Intervention Procedure

The final delivery model included: (1) completing the developmental monitoring form (SWYC) and support recommendation (CCD), (2) during wait time, (3) by the psychology trainees, (4) followed by integration in the routine pediatrician follow-up, capitalizing on the rapport and relationship between parent and physician while leveraging the on-going patient and family-centric initiative in the service line (Table 1).

**Table 1:** Outcomes, assumptions, need assessment, and interventions of the theory of change model.

**Domain**	**Outcome**	**Assumptions**	**Needs assessment**	**Intervention**
Reach & reaction: Will the intervention delivered reach the intended target groups with the right reaction?	Eligible children are reached.	Families of young children can be reached through trained staff. There is a demand for monitoring services to promote children's development. Families and pediatricians will accept the intervention.	Pediatricians do not have enough time in the clinic for developmental monitoring form administration. However, if the initial assessment is supported by another cadre, pediatricians can guide the families. The additional cadre needs to have time and relevant experience but cannot burden the existing staff. The country has a handful of developmental pediatricians with limited training opportunities in residency programs. The limited supply of professionals qualified in ECD is reflective of the low demand from parents of young children to monitor development. Hence, service in silos for developmental advice will not result in significant uptake. The pediatricians would not be invested in spending additional time or training effort on developmental consultation without due reimbursement. There has been significant value creation for psychosocial needs during the “*The Patient and Family-Centric Initiative”* for buy-in from physicians. Psychology clinic experience indicates families welcome discussion of emotional behavior problems and greater acceptance when reinforced by the pediatrician.	Partner with a psychology university and use psychology trainees. Provide training and clinical supervision. A form which provides greater false positives is preferable as no potential child and family in need is missed for a conversation about promotion of nurturing care. Utilize wait time for monitoring which is already a pain for the families. When wait times cannot be reduced, opportunities of utilizing it meaningfully to add value to overall experience can engage families. The service has to be free of cost to begin with. Personnel costs for psychology trainees can be reduced through training, clinical supervision, and credit for internship hours to administer the form. However, it has to be reinforced by the pediatrician capitalizing the trust and relationship. For the pediatricians it will be value added as they provide additional messaging on the health functioning of the patient within the same fee. Leverage the already in place “*The Patient and Family-Centric Initiative*” which values nurturing experiences as one of the core processes of care. Moreover, it is also now being recognized in the system that ECD is a long-term health outcome.
Capacity change: Will the intervention delivery and reach lead to the intended capacity changes?	Mothers acquire new capacity about nurturing care practices.	The advice will be understood and mothers will comply. Mothers have the capability to seek new knowledge. Mothers are motivated to improve the development of children.	Approach has to be simple with focus on milestones and also the environment. Pediatricians at AKUH are trusted as it is considered an elite hospital in the city. Families are generally from the middle class and educated. Schools heavily emphasize cognitive skills for entry which is a factor that can be heavily leveraged for engagement.	Use a simple checklist created for monitoring in the OPD with a focus on the role of the environment. This can help the parents reflect on their role in children's development. Reinforcement from the pediatrician is needed with focus on long-term achievement in school. This can help the parents reflect on their role in children's development.
Behavior change: Will the capacity change lead to the intended behavior changes?	Parents, specifically mothers, adopt new practices.	Mothers can make decisions about their child. Parents can observe improvement in child's development.	Generally, fathers have a greater role in decision-making. Generally, parents lack knowledge of developmental milestones.	Pediatricians reinforce both parents for uptake. Psychology trainees can provide guidance to parents for relevant websites or other sources for monitoring their children's development.
Direct benefits: Will the behavior changes lead to the intended direct benefits?	Children have greater opportunities to engage in play activities and interact meaningfully with parents.	Recommendations are mutually enjoyable and easy to implement. Families can afford play materials.	General parenting style is authoritative and less focused on nurturing interactions. Families come from the middle class and generally have access to toys and gadgets.	The recommendation should be based on enhancing interactions between the parent and child which can be inherently rewarding. Guide parents to sensible use of toys but also gadgets—which have become a part of families' lives.
Development changes: Will the direct benefits lead to the intended well-being changes?	Child's developmental outcomes improve.	Children have access to healthcare. Appropriate referral pathways exist for children in greater need.	Well-baby clinic visit does not include developmental monitoring as part of the core messages.	Reinforce completing scheduled well-baby visits. Include monitoring in every visit. Establish liaison with the developmental pediatrics department.

*AKUH, Aga Khan University Hospital; OPD, outpatient department*.

The developmental monitoring and support component was administered by a team of four: two research associates (psychology graduates) with significant experience with assessment of children with developmental disabilities and two psychology student trainees. Each student trainee was paired with an associate for quality assurance, and also supervised experience contributed toward their learning. As part of the procedure, the family was guided to see the trainee once the clinical assessment was completed by the nursing staff. The SWYC was completed during the wait time. Language (either English or Urdu) was chosen based on the family's preference, and trainees asked parents if they would like to fill the survey themselves or with the support of the trainee as an interviewer reading out the questions and tracking their responses for the parents. The raw scores on SWYC were calculated for a developmental milestones checklist covering cognitive, motor & language domains, emotional & behavioral symptom items, and family stress. The scores were then classified as “Appears to be meeting expectations” or “Needs review” based on the SWYC guidelines. This description was entered on a summary form attached to the patient file to be seen by the pediatrician. The follow-up reinforcement by the pediatricians was integrated as part of the routine consultation. Based on the SWYC summary form, they provided advice and recommended a referral if needed as reflected in the informal conversations with the nursing team assisting the physician. The physicians would usually sign a referral form or verbally inform the consultant they referred the child to which could either be a psychologist or a neurologist based on the nature of the issue. About 15% of the forms were re-evaluated by the first author with the families. Feedback was thereafter given to the trainees for any clarification or elaboration.

### Data Collection

Data from the parents were collected through a feedback form administered by the psychology trainees once the SWYC was completed. The parental feedback form comprised six questions, of which two were open-ended (“How did you feel before filling the form and after completing the [monitoring] session?” and “What did you like best about this mini-session?”) and four close-ended questions (“Do you think understanding your child's developmental progress and emotional needs is required?,” “Would you like to discuss the results of this form with your child's pediatrician?,” “Would you like further information on ways to stimulate your child, such as a brochure with guidelines for activities for various age groups?,” and “Have you ever visited a neurologist or psychologist before?” We could approach only the first 60 families for feedback. We could not continue due to logistic concerns in the waiting area and trade-off between collecting data on the developmental milestones and the feedback on the process. The former was deemed more important to make a case for integrating the service.

The psychology team members were asked to share their reflections as a qualitative written narrative shedding light on their experiences, perceived benefits to families, the physician response, and specific barriers and enablers to implementation or continuation of the service once the study ended. Physician feedback was obtained *via* email in response to findings shared with them about the developmental status of their patient population part of the study. The email was shared at two time points in the study: when one third of the sample was complete and when the study sample was completed. The data collection continued until the patient and family-centric initiative was in place.

### Data Analysis

The responses on the SWYC were scored based on the cut-off available with the form. Data were entered into an excel sheet by the trainees and were shared on a weekly basis with the first author. Frequency and percentages were calculated for children indicated at risk for further review by the pediatrician. Ongoing and overall trends were monitored as a team along with discussion of any challenges. The Bowen et al. (45) framework which describes the common focus areas of feasibility studies was used to evaluate the study across two areas: acceptability (How attractive, suitable, or satisfying was the intervention?) and implementation (How well was the study implemented as planned?). Data were analyzed in SPSS V22.0 for cross-sectional descriptive statistics. Qualitative feedback from parental experience and physicians and trainee reflections were analyzed using thematic analysis for an understanding of their experience from their respective perspective to inform scalability and hence scalability of the initiative. The analysis team included the ECD researcher (also Director Patient Experience of Care) and a developmental psychologist—who was independent of implementation to reduce bias. An inductive approach was employed for analyses following the standard procedures (46). The responses were coded independently by two team members for the qualitative data and then finalized in an agreement meeting. The codes were then broadly reduced under two aspects of feasibility, (i) acceptability defined as the extent to which the stakeholders perceived developmental monitoring and support to be attractive, suitable, and satisfying and (ii) implementation defined as how well the intervention could be implemented as planned within the available resources and what barriers and facilitators were identified.

## Results

A total of 182 families with 67 girls and 115 boys participated in the study. About 70% of families preferred administration in English. Additionally, 30% preferred to fill the form in themselves, and 70% asked the trainee to conduct the interview. The results indicate that 54% were detected as at risk on cognitive, physical, and language milestones and 76% were found at risk on emotional and behavioral symptoms requiring further advice about nurturing care and also referral (Table 2). Moreover, the trends indicated a greater number of positives in the developmental clinic as expected.

**Table 2:** Children identified for further review and referral.

**Developmental domain from SWYC**	**Identified for further review and referral-N (%)**			
	**Complex care**	**Acute care**	**Developmental care**	**Total**
	***N*** **= 24**	***N*** **= 129**	***N*** **= 29**	***N*** **= 182**
Cognitive, motor, and language milestones	10 (42)	64 (50)	24 (83)	98 (54)
Emotional and behavioral symptoms	13 (54)	89 (69)	23 (79)	138 (76)
Family questions	9 (38)	22 (17)	18 (62)	49 (27)

### Acceptability

Feedback to inform acceptability was collected from 49 of the total 60 families approached. Though interviews were intended for all, they could not always be conducted due to unavailability of families at times, e.g., if they were called in for the consultation. Parental feedback data from the 49 families indicated that 86% (*N* = 42) of parents felt understanding their child's developmental progress and emotional needs was important, 78% (*N* = 38) wanted to discuss the results of the form further with the pediatrician (3 parents said the decision to follow-up on the screening was at the pediatrician's discretion), and 73% (*N* = 36) of parents wanted further information in the form of a booklet for stimulation activities to conduct with their child, and only 1 had been to a pediatric neurologist or psychologist prior to the pediatric appointment.

Table 3 summarizes qualitative data from parents' feedback regarding acceptability across different domains: the content of monitoring items, overall feedback on the process, and the trainee. Most parents regarded the monitoring session as a “*good initiative*” as it helped them understand the significance of their role in their child's development and an increased awareness about developmental milestones and emotional needs in general. One of the families said “*I was surprised to know that emotions of parents play a major role in the child's life”* while another appreciated the session saying “*In the beginning we thought the questions would be irrelevant, but in the end we realized that these are important questions that will help the child in the future.”* A family shared their interest in having more of these sessions: “*It was relatable, I would like more of such sessions.”* A few parents enjoyed thinking about and answering family risk-related questions: “*[I appreciated] the personal family questions.”* Two parents also reported that completing the form made them feel the hospital was concerned about their child's health and well-being.

**Table 3:** Parental qualitative feedback regarding the screening process.

**Content of the monitoring form**
[I liked] the questions related to the spouse/partner (F5, 4 months, GP). [I liked] the questions related to the spouse/partner (F12, 13 months, GP). [I liked] the questions related to the spouse/partner (F21, 2 months, Cardio). [I appreciated] the personal family questions (F43, 35 months, Cardio). [I appreciated] questions about the child's sleeping habits (F15, 6 months, GP). [I appreciated] questions that asked whether the child is involved in fights (F59, 60 months, GP). …it was good you asked about the relationship of parents with the child (F3, 32 months, GP). I was surprised to know that emotions of parents play a major role in the child's life (F24, 8 months, GP). I think I gained new information from the questions (F1, 19 months, GP). I liked the questions related to emotional changes with a new baby (F17, 16 months, Cardio). I liked the questions related to emotional changes with a new baby (F20, 2 months, GP). [The questions] helped me recall important things about my child (F9, 13 months, GP). [The questions] helped me recall past memories about my child. (F31, 12 months, GP). The questionnaire is very comprehensive (F2, 2 months, GP). The questionnaire highlighted important points that usually parents would ignore (F25, 61 months, GP). I didn't know these questions were real issues (F35, 7 months, GP). In the beginning we thought the questions would be irrelevant, but in the end, we realized that these are important questions that will help the child in the future (F59, 60 months, GP). The conversation highlighted important points [about our children] that we usually ignore (F25, 61 months, GP).
**The monitoring process (during the waiting period)**
It was relatable, I would like more of such sessions (F4, 11 months, GP). It was good. There should be more activities like this one (F34, 60 months, GP). You concluded and connected the behavior of the child with the parents (F3, 32 months, GP). You connected my current situation with my wife and children, I really appreciate that (F38, 7 months, Cardio). This would help a parent whose child is suffering (F8, 14 months, GP). So many things are cleared up that we were stressed out about (F52, 4 months, GP). The hospital is very interested in the development and mental health of a child. It was good, laughed a lot, made us feel good (F39, 53 months, Cardio). [It seems] the hospital is concerned about us (F51, 49 months, GP). This was something new related to children (F40, 60 months, GP). Very useful, would want to continue such sessions in the future (F3, 32 months, GP). We felt like we know our children better after this conversation (F54, 30 months, GP).
**The trainee**
It was really nice talking to you (F38, 7 months, Cardio). It felt good to talk to you (F57, 10 months, GP). Your behavior with us was good (F51, 17 months, Cardio). I'm satisfied after meeting you, it felt good to talk to you (F56, 12 months, GP).

*Cardio, Cardiology; F, Family; GP, General Pediatrics*.

The patient experience team shared data about the families' responses on the SWYC and the number of children screened at risk with the pediatricians for their reflections. Two pediatricians formally responded with comments about their patients' outcomes and parental satisfaction. One physician (complex care physician) was interested in further exploring why 50% of his screened patient population was found to be at risk for delayed milestones, with 61% at risk of developing neurotic symptoms and 75% at risk for either reason. His response to this information was “*75% is a huge number, why do you think that is the case? Is it a selection bias - these kids are sick with chronic diseases and that is why they are at risk? Is there any correlation with complexity of disease and risk? Maybe an analysis of that will be insightful*.” Another physician (acute care specialist) reported, “*I am happy to assist. You can continue with my outpatient and inpatient [patient population]. This is excellent and amazing data. I would suggest continuing this*.” The developmental psychologist felt it was helpful to have the parents complete a form before the consultation saying “*It sets the tone, and the conversation becomes easier in the clinic. Moreover, having some sort of screening makes the parents feel it is an objective assessment compared to just clinical observations. It also saves time*.” When asked about the role of trainees, her view was that it can be a great way of exposing trainees to the field of ECD: “*There is no formal teaching in place for developmental problems in young children. Having trainees and supervising them can be a way of hands-on training and exposing them at the right time when they are about to begin their careers*.” A similar view was shared by one of the trainees, “*This service should have trainees because it's a win-win situation for both sides as trainees need experience and obviously, they will learn a lot, and this service can't be handled by one person only, so having trainees is a sustainable idea. It's like, the more the merrier because more trainees, more surveys done in less time and accurate results”* (Trainee 2).

All four team members including trainees shared their reflections and indicated they found the process helpful for themselves, aiding their counseling skills. One of them said: “*I learnt a lot. Like. different milestones, items regarding autism, at [the] same time to assess parental stress. which help[s] us in parental counseling*…” (Associate 1). Another one shared that interacting with families was something she enjoyed the most: “*One thing I loved the most was the clinical experience. I got to meet and interact with patients directly*” (Trainee 2). A student trainee expressed that the experience had inspired her to pursue a career as a child psychologist: “*I implemented my bookish knowledge in real-life scenarios which made clear that child development is the path I would love to [choose] for my further studies*” (Trainee 1). The student trainees were also provided with an opportunity to present the study findings in a departmental research event which was an additional motivating factor: “*I also got a chance to take part in [the] research poster review. The experience taught me how to present and defend my findings*” (Trainee 1). When student trainees were asked if this project benefited them in anyway, one of the trainees expressed, “*Yes, as a student, I learned the importance of milestones myself. This project made me more conscious about delayed milestones that I often ignored in the children of my friends and family. I also realized that post-partum depression is something in which I should work on in future. I also learned that a healthy bond between the husband and wife is very essential for their child's healthy mind”* (Trainee 1).

The significance for the families was also felt by the trainees: “*A quick screening like OPD screening helps us to guide parents properly, it helps us to refer children to concerned people according to the child's problem”* (Associate 1). Another trainee felt the process was kind of relieving for families: “*Monitoring in the OPD is challenging but it's important. We need to continue with the monitoring. Parents felt better when we spoke to them about their child's behaviors especially those stressed due to the child's illness*” (Associate 2). Another trainee shared similar observations while interacting with the families, “*When families got a friendly person to talk in clinic, they opened up to us easily and most of them talked their heart out which made them feel good”* (Trainee 2).

### Implementation

Practical challenges related to constrained resources and clinical referral pathways were identified over the course of the study by the trainees conducting the screening. Firstly, while all staff were cooperative, they were occupied in multiple duties, so occasionally forgot to share information on study-eligible patients with the trainee for SWYC assessment, “*The obstacles I faced. Like, unable to figure out how to find the family that we need[ed] to [interview], so basically the system needs to be changed a bit, so that no parent is missed during the process.”* (Trainee 1) as mentioned by one of the trainees as well when asked how this service can improve. Another trainee reported time as a challenge, “*Some of the physicians didn't value what we did so they used to ask us to do our survey after their consultation and others used to give us time to talk to the patients”* (Trainee 2). Secondly, the trainee had to carry 12 versions (relevant to different age groups) of printed SWYC forms and identify the correct form in a limited time period and constrained space, while conversing with waiting parents and children; identifying the correct form based on the child's age was made more difficult by parents giving vague or incorrect answers about the child's precise age. Out of all the methods that were used to assess SWYC with the client's parents, it was found to be most convenient when dedicated space was available, “*The only improvement I think it needs is the time management and I guess a proper room where we can do our survey without any distraction”* (Trainee 2) as reported by one of the trainees. When requested for recommendations two of the trainees felt physician understanding could be improved, “*Physicians need awareness training about developmental and emotional problems. I also think there need[s] to be more developmental psychologists available in the out-patient [clinic] for individual counseling”* (Associate 1). Another student trainee shared similar feedback after interacting with the pediatricians, “*The ones with whom I interacted; they were more than happy to receive the score sheet. There were times when they were amused to see the contrary results. For example, there were [a] couple of times when we shared the results with the doctors, they did not realize that a child's milestones w[as] not fully achieved, or the parents need[ed] counseling, or the mother [wa]s going through post-partum depression. These were some of things which [a] few of the physicians did not notice, but were surprised to see such results. Due to lack of time, doctors were mainly focusing on the problem that the parents brought, rather than observing the parents”* (Trainee 1).

## Discussion

The findings from this study indicate that developmental monitoring with support for families was largely acceptable to families and trainees with evidence of preliminary operational feasibility. A greater number of children indicated a need for intervention for the behavior symptoms compared to other developmental domains. Another observation from the data was that of parents whose children were put forward for further review, many were not concerned about their child's milestones and therefore had never consulted any physicians. It could also be due to the fact that mild cases may go unnoticed by parents and also by physicians (47). A similar observation from Pakistan has been highlighted by Mushtaq and Rehman (48). These findings have implications for early intervention and support for children at risk of developmental difficulties but also for those who can benefit from general parenting advice. It also creates the opportunity to provide a vision for ECD in healthcare beyond disability to optimal development pertaining to all children through provision of nurturing care (49). Developmental status is indeed one of the key indicators of long-term health outcomes beyond survival (50). A few parents also appreciated the items related to the family environment as helpful. As the program grows and pediatricians feel more confident, this form may be an opportunity to touch upon the family environment which can be a stress factor affecting the health and development of young children. The addition can be valuable as dedicated family services do not exist in the country.

Feedback from one of the pediatricians and some of intervention team members suggested a need for greater understanding and discussion between ECD professionals and pediatric consultants with regards to the data on SWYC outcomes. This feedback was from a pediatrician who dealt with chronic conditions and perhaps required more information that could help create an integrated care plan. One recommendation can be to involve a developmental pediatrician in future as part of the team who also understands the health needs along with development. Another pediatrician who dealt with acute care issues felt it was a great addition to his ongoing clinic. The developmental psychologist had an interesting perspective with respect to not just the service but also how utilizing trainees could address training of the next generation of psychologists for early development. Unfortunately, we could not conduct interviews with pediatricians but the difference in feedback from the three pediatricians seems to be due to the fact that they were dealing with different patient populations. It also highlights that developmental monitoring and support can have a different value to ongoing services for acute and chronic health issues.

Psychology trainees were important stakeholders as they were envisioned to be the key delivery staff for future scale-up. Their feedback was encouraging, and they enjoyed the experience. We think it is because the health center is an elite prestigious center and hence valued by the trainees for their future career aspirations. We ensured that they were supervised and hence were paired with psychology associates with considerable experience in developmental assessments. Regarding the larger feasibility of the intervention, the feedback from the trainees highlighted aspects related to survey administration while no feedback was received from physicians. One reason could be that a family-centric initiative was ongoing for about 2 years before the roll-out of this study. Hence, we did not face any challenges upfront in physician engagement or buy-in for the idea to move to a family-centric approach. We also believe successful demonstration of feasibility lies in designing the intervention as value added for all stakeholders. That was possible because the team spent considerable time designing the ToC, laying out all opportunities and risks. Additional effort was designed for the risks. A meta-analysis of home visiting programs to prevent child neglect and abuse found that programs with a clear ToC with intervention components aligned to population needs had a higher chance of success (51). The intervention had value for families and pediatricians but also psychology trainees who got an opportunity to learn and also contribute toward child health. In the long-run, the hospital benefits from improved services in terms of patient and family experience (52). Moving forward on the journey from invention to social innovation at scale, initiators should make a conscious effort to leverage partnerships between key stakeholders to achieve optimal development for children (53, 54). Effective implementation of partnerships between public and private health sectors can be achieved through a robust ToC entailing creating partnership norms, crafting collaborative work plans, conducting regular audits, and evaluation using such tools as the Partnership Assessment Tool (55).

The strengths of the study include a cost-effective design at the outset to leverage existing resources and context-specific strengths. The trainees were interns/associate psychologists from a local psychology university seeking capacity building for clinical training. Studies from healthcare have found positive benefits of volunteers on patient experience (56). Parental trust in pediatricians and parental perceptions regarding the credibility of the study site as a teaching hospital were utilized to enhance feasibility. Another strength was that the study was implemented as part of an on-going patient and family-centric initiative emphasizing compassionate care. This allowed for a quick buy-in of the physicians whereby they were aware of the elements of psychosocial care and effect on health outcomes. There were several limitations of the study. One, the SWYC is not validated for the Pakistani population. However, the SWYC was intended to be used as an indicator of need for parental conversation by the pediatrician. In case of due concerns, children and their parents were then referred to developmental specialists. A study using SWYC in the Brazilian context found a similar performance of children between birth to 36 months as North American children (57). Moreover, the authors felt given the scale of the problem, this limitation of validated tools in the Pakistani context should not be a barrier to initiation of developmental monitoring processes. Secondly, we could not collect feedback from all the families about their experience with the intervention process nor could we approach physicians through in-person conversations for their insights about the services owing to resource constraints. An additional limitation is that for the purpose of this study, we were not able to follow up with parents and children to collect data on how many referred/identified at-risk children went on to connect with specialist services for additional support and if there was any agreement on the clinician diagnosis and screening results. However, the primary purpose of the initiative was to help the parents become aware of early developmental milestones and what their role could be in a manner that was acceptable. Since relatively educated families use the services at the hospital, we felt the increased awareness in itself could be an intervention acting as a nudge for the families in every outpatient visit for their child (in the first 5 years of his/her life). Several children with indicated need were referred as noticed through informal conversations but a systematic record could not be maintained. Also, there was no protocol in place in the service for referral of children with identified developmental and emotional needs. Decisions were usually based on the physician's preferences of services and providers, and creating a protocol was beyond the scope of the study. It was also not feasible for the research team to follow-up with the physicians to understand how the results were discussed with the families and if they developed distress. Given that pediatricians have an on-going relationship with families, we assume that may have mitigated some of the risks. Finally, two of the authors were also part of the consultant team and their reflections could be biased. Hence, the data analysis team included a developmental psychologist independent of the intervention team.

### Future Direction

Further suggestions and key feasibility findings for the intervention are summarized in Table 4. A key recommendation is to continue the model with relevant changes suggested by the feasibility findings and to subsequently test the model using a robust evaluation strategy while ensuring adequate resources. Continued leadership buy-in and support will be important to sustain engagement of the physicians for family-centric care. Though feedback was received from only three consultants it had made us realize that the intervention model has to be co-designed in the next phase and tailored to the different sets of patient needs to maximize benefits. Moreover, developmental outcomes will need to be included as a key indicator of patient-reported health outcomes for continued improvement in quality of services. We conclude that when designing implementation models for developmental monitoring, the context needs to be carefully considered for feasibility and should include iterative learning cycles for continuous improvement. Due time and effort should be invested to understand how the intervention would operate but also how it would lead to a behavior change.

**Table 4:** Future directions.

**Theme**	**Domain**	**Recommendations and implications**
Operational	Communication gap between administrative staff and developmental trainee	Parents/patients can connect with the trainee at the time of registration at the clinic reception
	Managing paper copies of 12 different age-group forms was cumbersome. Parents had various language preferences. Some parents preferred to be interviewed, while others were comfortable completing the SWYC questions themselves.	An app for use on a tablet or smartphone can be developed for auto-calculation of the patient's exact age and identification of the appropriate screening form in the respondent's preferred language. This app can be designed for surveys/questionnaires that can be completed by the parents and by the trainee. For parents with lower literacy levels, the app can include an audio option (read out loud the survey questions) or speech to text and text to speech options.
	Limited space in clinic, with no toys/books/play area/material available for waiting children	Dedicated space for screening and counseling with parents is required, which can also help address patient privacy concerns, while providing children resources to play with while their parents are engaged in the screening process.
	Ensuring leadership buy-in.	Ensure leadership willingness for continued services. In addition, all the staff members, that includes doctors, nurses, and administration staff, etc. should be briefed about the purpose of the QI of this questionnaire for their engagement. Should also be communicated to families as a new meaningful initiative.
Technical	Communicating news about developmental risks to parents, particularly when parental knowledge is low at the outset of screening	It was observed that while parents reported being satisfied with their child's current behavior and development, some were usually unaware that their child could be at risk. Parental education and counseling by the pediatric consultant needs to be sensitive to parental distress that can be caused when communicating results. Training for the pediatricians needs to be incorporated.
	Physician engagement is limited due to time constraints	Structured monthly or bi-monthly meetings are required to share trends, challenges experienced and addressed by physicians and the Patient Experience team. Include a developmental pediatrician in the team.
	Limited trained/qualified human resource	Dedicated staff are required to counsel and screen the parents/patients; pediatric residents can be trained in-house. Additionally, collaborations with partner universities can encourage internships for medical students or psychology/allied health students to complete the screening and provide a training opportunity simultaneously. Maintain a liaison with the university and share feedback about student progress. This enhances engagement of the students and ensures professionalism.
	Engagement of stakeholders	Ensure added value to engage all stakeholders: parents, physicians, leadership. Regular meetings and streamlined communication between pediatricians, developmental specialists, and hospital administration can improve referral pathways from primary to specialist care, while incorporating parental feedback into these processes.
Research	Evaluation	Evaluation of families' knowledge, attitude, and practices (KAP) about early childhood development should include a randomized sampling approach. It will be important to capture their waiting experience, and the data can be used for leadership buy-in. Physician and trainees KAP are also an important set of process outcomes. Moreover, follow-up of children connecting with services should also be considered through a follow-up call. Qualitative data from parents also need to be ensured for greater insights about the process.
	Intervention	Co-design the intervention with different disease specialties. Start slow, ensure bottlenecks are ironed out and follow a phased roll-out with different specialties. Ensure implementation is evaluated for fidelity, acceptance, demand, and use of services.

*QI, Quality Improvement; SWYC, Survey of Well-being of Young Children*.

## Data Availability Statement

The raw data supporting the conclusions of this article will be made available by the authors, without undue reservation.

## Ethics Statement

The studies involving human participants were reviewed and approved by Ethics Review Committee at the Aga Khan University as an exemption. Written informed consent for participation was not required for this study in accordance with the national legislation and the institutional requirements.

## Author Contributions

MR conceptualized and designed the study, trained volunteer psychologists to implement the intervention in the outpatient clinic, prepared analysis plan, and led the writing of the manuscript draft. WM analyzed and interpreted the study findings and contributed to the manuscript draft. KE contributed to the analysis of family experience data and contributed with reflections on her experiences as an assessor in this program. BH provided intellectual input toward the study design in his capacity as Pediatrics service line chief and to the manuscript drafts. All authors contributed to the article and approved the submitted version.

## Conflict of Interest

The authors declare that the research was conducted in the absence of any commercial or financial relationships that could be construed as a potential conflict of interest.

## Publisher's Note

All claims expressed in this article are solely those of the authors and do not necessarily represent those of their affiliated organizations, or those of the publisher, the editors and the reviewers. Any product that may be evaluated in this article, or claim that may be made by its manufacturer, is not guaranteed or endorsed by the publisher.
